# Cervical lymph node enlargement as the initial manifestation of rectal cancer

**DOI:** 10.1186/s12876-021-01628-5

**Published:** 2021-02-09

**Authors:** Tong-Hui Xie, Peng Su, Jian-Guo Hong, Hui Zhang

**Affiliations:** 1 Department of General Surgery, Qilu Hospital of Shandong University, 107 West Wen Hua Road, Jinan, 250012 Shandong China; 2 Department of Pathology, Qilu Hospital of Shandong University, 107 West Wen Hua Road, Jinan, 250012 Shandong China

**Keywords:** Colorectal cancer, Lymph node metastasis, Surgery

## Abstract

**Background:**

Colorectal cancer is a very common malignant tumor worldwide. The clinical manifestations of advanced colorectal cancer include the changes in bowel habits, hematochezia, diarrhea, local abdominal pain and other symptoms. However, the colorectal cancer with an initial symptom of cervical lymph node enlargement is extremely rare. In this article, we report a case of rectal cancer presenting with cervical lymph nodes enlargement as the initial symptom.

**Case presentation:**

A 57-year-old woman was admitted to our hospital for cervical lymph node enlargement which was accidentally detected during physical examination. Computed tomography scan revealed multiple enlarged lymph nodes in the neck. Cervical ultrasound showed normal thyroid gland and multiple left supraclavicular lymph nodes enlargement. The patient underwent lymph nodes biopsy and pathologic results showed metastatic adenocarcinoma. The subsequent lower gastrointestinal endoscopy revealed a mucosal bulge lesion located at rectus and biopsy revealed adenocarcinoma. The patient underwent rectal cancer resection. She is alive with no evidence of recurrence or new tumors 2 years after surgery.

**Conclusions:**

Cervical lymph node metastasis is a rare metastatic way in colorectal cancer. This is the first case of rectal cancer presenting with cervical lymph nodes metastases as the initial symptom. Surgical resection combined with postoperative chemotherapy improved long-term prognosis of the patient. This rare metastatic way of rectal cancer should be paid attention for clinicians.

## Background

Colorectal cancer is the third most common malignant tumor and the second leading cause of cancer-related death in the world, with more than 1.4 million new cases and 0.8 million deaths each year [1]. Lymph node metastasis in patients with colorectal cancer is common, indicating that the tumor is at least in the middle stage [2]. In general, the lymph nodes near the primary site are the most common metastatic site for rectal cancer [3]. In this article, we report a case of rectal cancer presenting with cervical lymph nodes enlargement as the initial symptom. This patient had no other symptoms and the skip metastasis was the main form of metastasis for this case.

## Case presentation

A 57-year-old woman was admitted to our hospital for a cervical mass which was incidentally detected during physical examination. She presented no abdominal pain, abdominal distension or gastrointestinal bleeding. Physical examination revealed multiple cervical masses with the maximum diameter of 3 cm. The masses were hard and presented poor mobility.

**Fig. 1:**
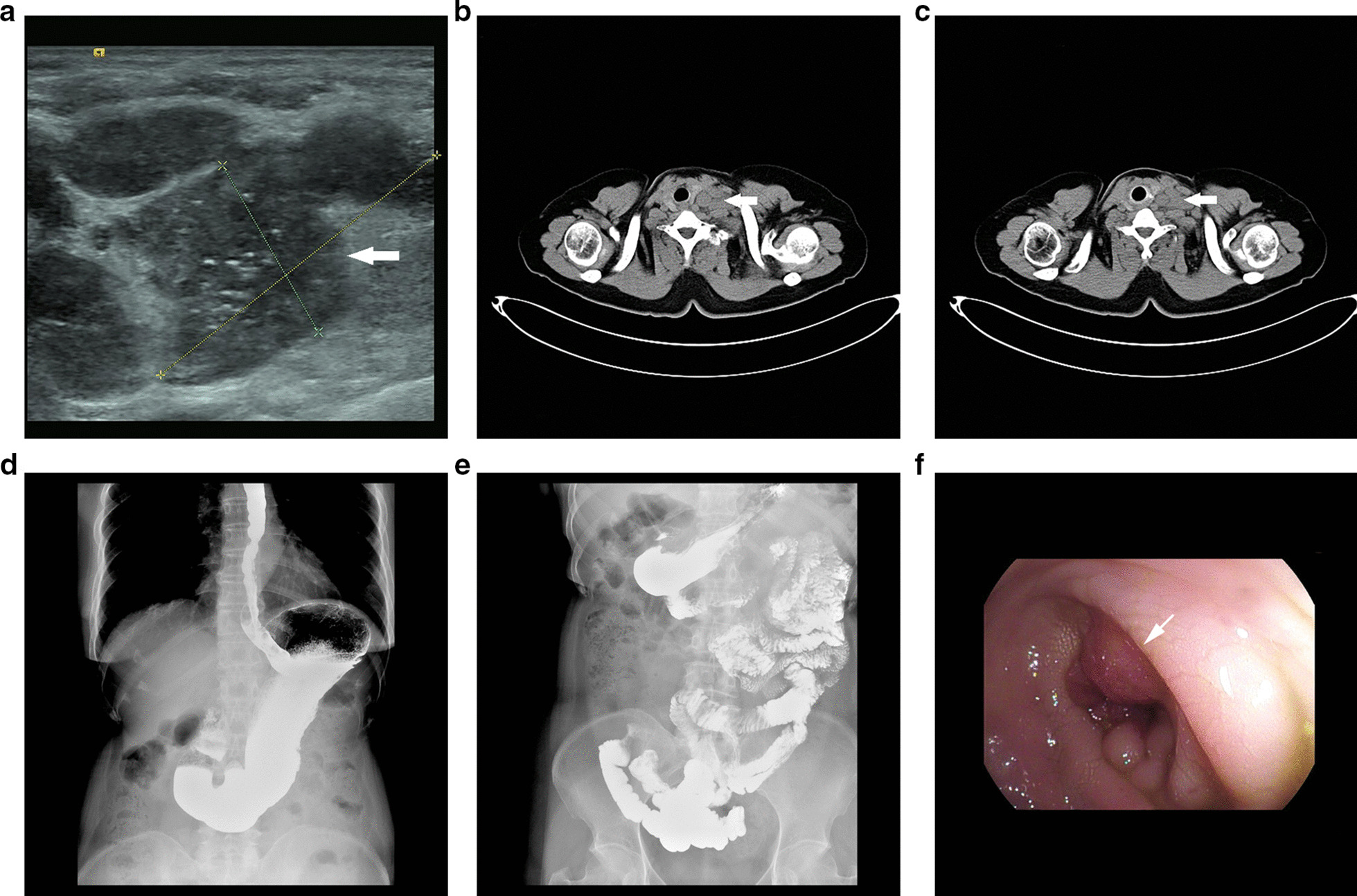
**a** (Cervical ultrasound) showed multiple hypoechoic nodules in the left neck and left supraclavicular fossa with clear boundaries, irregular morphology, multiple dotted strong echoes and rich blood flow signals. **b**, **c** (Chest computed tomography) showed multiple enlarged lymph nodes in the bilateral submandibular region, bilateral carotid sheath and intermuscular space of the cervical root. **d** (Whole gastrointestinal tract barium X-ray radiography) showed whole gastrointestinal tract has no sign of obstruction. **e**, **f** (Lower gastrointestinal endoscopy) showed irregularly elevated rectal mucosa with congestion, erosion and easy bleeding. The lesion caused rectal narrowing and the endoscopy was not able to pass through

Laboratory examination showed elevated levels of CEA (15.59 ng/ml, normal range, 0–5), CA19-9 (42.78 ng/ml, normal range 0–37) and CA242 (22.50 U/ml, normal range 0–20). Serum levels of CA125, CA15-3 and AFP were within the normal range. The complete blood count, thyroid function and liver function were normal. Cervical ultrasound showed normal thyroid gland and multiple left supraclavicular lymph nodes enlargement with the maximum diameter of 3.3 cm (Fig. 1a). The patient underwent chest computed tomography (CT) and whole gastrointestinal tract barium X-ray radiography. The CT scan revealed multiple enlarged lymph nodes in the bilateral submandibular region, bilateral carotid sheath and intermuscular space of the cervical root (Fig. 1b, c). Whole gastrointestinal tract barium X-ray radiography revealed no obvious lesion (Fig. 1d, e).

**Fig. 2:**
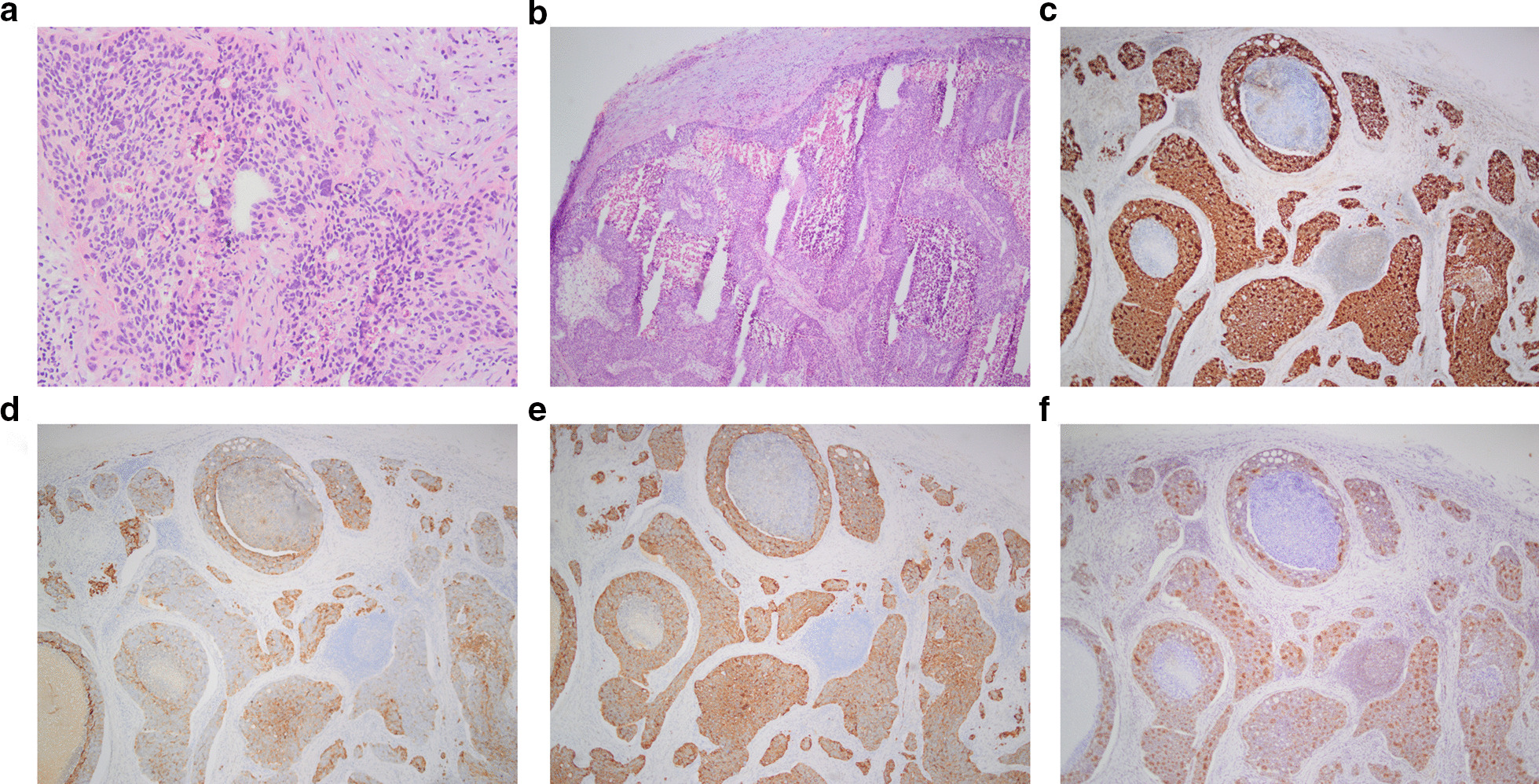
showed the pathologic results of the cervical lymph nodes biopsy. **a** (HE staining, × 40) and **b** (HE staining, × 200) showed metastatic poorly differentiated adenocarcinoma was found in lymph nodes with marked atypia arranged in nests and cribriform pattern. Necrosis and scattered calcification were present in the center of the carcinoma. The tumor cells are heterogeneous with pathological mitotic figures. Based on the histological morphology, the adenocarcinoma most likely arose from breast or gastrointestinal tract. Immunohistochemical examination showed that the neck mass was positive for GCDFP (**c**, × 40), CK19 (**d**, × 40), CK20 (**e**, × 40) and CDX-2 (**f**, × 40)

The patient underwent lymph nodes biopsy. Intraoperative frozen section analysis showed metastatic adenocarcinoma cells with marked atypia arranged in nests and cribriform pattern within the lymph nodes. Necrosis and scattered calcification were present within tumor foci (Fig. 2a, b). Based on the histological morphology, the metastatic adenocarcinoma most likely arose from breast or gastrointestinal tract. Given that a mammary nodule was detected on palpation, resection of the mammary nodule was performed during this operation. However, intraoperative frozen section analysis showed mammary gland hyperplasia. After the operation, immunohistochemical examination of the cervical lymph nodes biopsy was performed and showed that the tumor was positive for GCDFP (Fig. 2c), CK19 (Fig. 2d), CK20 (Fig. 2e) and CDX-2 (Fig. 2f), but was negative for actoglobulin, ER, PR and TTF-1.

After the operation, the patient underwent upper gastrointestinal endoscopy but no esophageal or gastric tumors were detected. In order to identify the primary tumor location, pelvic enhanced CT scan was performed and it revealed uneven thickening and enhancement of the rectal wall. The subsequent lower gastrointestinal endoscopy revealed a mucosal bulge lesion located at rectus (Fig. 1f) and biopsy revealed rectal adenocarcinoma. The patient underwent rectal cancer resection. During the operation, we found that the tumor was located at rectum above the peritoneal reflexion. The tumor was hard and the size was about 3 × 4 cm. The rectal tumor penetrated rectal serosa and invaded the surrounding adipose tissue. Metastatic adenocarcinoma was detected within peri-intestinal lymph nodes. Based on intraoperative exploration and previous medical history, rectal cancer resection was performed and the patient recovered uneventfully. Eventually, based on the patient's postoperative pathologic results, the tumor was diagnosed at stage IV A (T3N2bM1a). The patient recovered uneventfully and no evidence of recurrence or new tumors was found during 2 years follow-up.

## Discussion and conclusions

Colorectal cancer is the third most common malignant tumor and the second leading cause of cancer death worldwide [4]. Direct invasion, hematogenous spread, lymphatic spread and implantation metastasis are the principal ways for metastasis in colorectal cancer. Although most malignant lymphadenopathy in the neck represent lymphomas or metastases from head and neck primary tumors, occasionally, metastatic disease from remote sites presents as cervical lymphadenopathy with or without an obvious primary tumor [5]. The case of rectal cancer reported in this article presented enlarged cervical lymph nodes as the initial symptom without any signs or symptoms of primary organ involvement is extremely rare.

Gastrointestinal tumor metastases in the left supraclavicular lymph node, also known as the Virchow lymph node, is often the sign of advanced malignant disease (stage IV). In 1848, the German pathologist Rudolf Virchow first described it as an enlarged gland associated with gastric cancer. Virchow’s node has come to be known as a signal node, signaling the presence of an underlying cancer form a primary lesion in the upper abdomen [6]. The most common digestive tract tumor with supraclavicular lymph node metastasis is gastric cancer. Cervical lymph node metastasis is an extremely rare metastatic way for colorectal cancer. The most common metastatic sites for colorectal cancer were regional lymph nodes and liver. Other sites include lung, peritoneum, ovary, central nervous system, bone, kidney and uterus. Adrenal gland, hilar lymph node, skin and muscle are the extremely rare metastatic sites for colorectal cancer [7].

At present, the mechanism of distant non-regional lymph nodes metastases in patients with colorectal cancer remains unclear. According to previous reports [7], skip micrometastasis between regional lymph nodes can be seen in 18% of cases. This case we reported would contribute to the interpretation of the mechanism of skip metastasis. For postoperative pathology, CDX-2 and CK20 are the most common immunohistochemical type in colorectal cancer [8]. CDX-2 is expressed in more than 80% of colorectal cancer. Expression of CK20 combined with CDX-2 can improve the diagnostic accuracy of colorectal cancer [9]. The positive rate of CK19 in gastric cancer is higher than that in colorectal cancer. GCDFP is positive in breast cancer with high specificity but low sensitivity [10].

For patients with supraclavicular lymph node metastasis, a combination of surgical resection and appropriate radiochemotherapy can achieve a good prognosis. According to previous reports, postoperative chemotherapy with FOLFOX regimen can improve the prognosis. The advantages of 18 Fluorine-labeled 2-fluoro-2-deoxy-D-glucose positron emission tomography (FDG-PET) in identifying the primary site and determining the correct diagnosis have been proved [11]. A multidisciplinary team is recommended in determining the diagnosis and establishing the treatment strategy for patients with cervical lymph nodes metastases. The development of distant lymph nodes metastases represents advanced disease and therefore, the main purpose of treatment is to improve the quality of life.

## Data Availability

Data sharing is not applicable to this article as no datasets were generated or analyzed during the current study.

## Abbreviations

CT: Computed tomography
FDG-PET: 18 Fluorine-labeled 2-fluoro-2-deoxy-d-glucose positron emission tomography
CEA: Carcinoembryonic antigen
CA19-9: Carbohydrate antigen 19-9
CA24-2: Carbohydrate antigen 24-2
CA125: Carcinoembryonic antigen 125
CA15-3: Cancer antigen 15-3
AFP: Alpha-fetoprotein
